# Water Developments and Canids in Two North American Deserts: A Test of the Indirect Effect of Water Hypothesis

**DOI:** 10.1371/journal.pone.0067800

**Published:** 2013-07-02

**Authors:** Lucas K. Hall, Randy T. Larsen, Robert N. Knight, Kevin D. Bunnell, Brock R. McMillan

**Affiliations:** 1 Department of Plant and Wildlife Sciences, Brigham Young University, Provo, Utah, United States of America; 2 Department of Plant and Wildlife Sciences and The Monte L. Bean Life Sciences Museum, Brigham Young University, Provo, Utah, United States of America; 3 Environmental Programs, United States Army Dugway Proving Ground, Dugway, Utah, United States of America; 4 Utah Division of Wildlife Resources, Salt Lake City, Utah, United States of America; Bangor University, United Kingdom

## Abstract

Anthropogenic modifications to landscapes intended to benefit wildlife may negatively influence wildlife communities. Anthropogenic provisioning of free water (water developments) to enhance abundance and distribution of wildlife is a common management practice in arid regions where water is limiting. Despite the long-term and widespread use of water developments, little is known about how they influence native species. Water developments may negatively influence arid-adapted species (e.g., kit fox, *Vulpes macrotis*) by enabling water-dependent competitors (e.g., coyote, *Canis latrans*) to expand distribution in arid landscapes (i.e., indirect effect of water hypothesis). We tested the two predictions of the indirect effect of water hypothesis (i.e., coyotes will visit areas with free water more frequently and kit foxes will spatially and temporally avoid coyotes) and evaluated relative use of free water by canids in the Great Basin and Mojave Deserts from 2010 to 2012. We established scent stations in areas with (wet) and without (dry) free water and monitored visitation by canids to these sites and visitation to water sources using infrared-triggered cameras. There was no difference in the proportions of visits to scent stations in wet or dry areas by coyotes or kit foxes at either study area. We did not detect spatial (no negative correlation between visits to scent stations) or temporal (no difference between times when stations were visited) segregation between coyotes and kit foxes. Visitation to water sources was not different for coyotes between study areas, but kit foxes visited water sources more in Mojave than Great Basin. Our results did not support the indirect effect of water hypothesis in the Great Basin or Mojave Deserts for these two canids.

## Introduction

Anthropogenic manipulations to landscapes or resources that are designed to benefit wildlife may have negative indirect effects on wildlife communities [1]–[3]. Indirect effects from anthropogenic manipulations are likely most pronounced when alteration influences resources that are limiting. In arid regions, water available for drinking (hereafter free water) is a limiting resource for some species that is often manipulated to increase distribution or density of animals [4]–[7]. Despite the widespread and long-term manipulation of free water (e.g., water developments for wildlife and livestock), there is little supporting information and much controversy concerning how this anthropogenic manipulation influences native species [8]–[10]. Water developments may be beneficial for some native species [11]–[13]. For example, water developments sustained suitable habitat for bighorn sheep (*Ovis canadensis*) where natural sources of free water were in decline [14]. However, manipulating a limiting resource, such as free water, may impose negative indirect effects on native species that have adapted to minimal availability of that resource.

The manipulation of free water in arid landscapes (i.e., addition of water developments) potentially weakens the advantages that arid-adapted species have accrued to minimize interspecific competition and predation from species that are water-dependent. In the Great Basin Desert, for example, it has been argued that water developments remove the limitation of arid systems to coyotes (*Canis latrans*) which compete with kit foxes (*Vulpes macrotis*) for habitat, space, and food [15]–[17]. This association is largely based on the differential physiological demand of free water by coyotes and kit foxes. To obtain enough preformed water (available in prey items) to survive in the absence of free water, both coyotes and kit foxes need to consume more prey than required to meet energetic demands [18]. However, coyotes need to consume twice the amount of prey per unit of mass relative to kit foxes to acquire sufficient preformed water to survive without free water creating an advantage for kit foxes in arid landscapes [18]. Furthermore, coyotes depend on evaporative cooling to expel heat and therefore have higher rates of water loss compared to kit foxes that rely on thermal conductance which reduces water loss [19]. Kit foxes also adhere more strictly than coyotes to behavioral adaptations that minimize water loss such as subterranean living and nocturnal activity [18], [20]. Thus, it is less energetically feasible for coyotes to inhabit areas that lack free water relative to kit foxes. Water developments may, therefore, indirectly affect the arid-adapted kit fox by enabling the water-dependent coyote to occupy an otherwise inhospitable system and exert asymmetric interspecific competition on kit foxes (i.e., indirect effect of water hypothesis) [15], [16].

The indirect effect of water hypothesis is comprised of two testable predictions: water-dependent competitors 1) will occur more frequently in areas near free water and 2) will spatially and/or temporally displace subordinate competitors. The predictions of the indirect effect of water hypothesis, however, have not been formally evaluated for canid communities and it is unclear whether this hypothesis is broadly applicable in arid systems. Our objective was to test the indirect effect of water hypothesis in the Great Basin and Mojave Deserts using coyotes and kit foxes as a model community. Specifically, we 1) evaluated support for the two predictions of the indirect effect of water hypothesis and 2) assessed relative use of free water by coyotes and kit foxes. This information will provide new insight into how anthropogenic modification of landscapes and resources may influence interspecific interactions and community dynamics.

## Methods

### Ethics Statement

Fieldwork was approved and sanctioned by the United States Department of Defense and the Utah Division of Wildlife Resources. Permission to access land in Great Basin was obtained from the United States Department of Defense (United States Army Dugway Proving Ground). Permission to access public land in Mojave was not required.

The kit fox is a protected species and we did not handle any individuals. Thus, our study was strictly observational in nature (using infrared camera traps) and no specific permits or regulations for animal care were needed according to the Institutional Animal Care and Use Committee of Brigham Young University.

### Study Areas

This study was conducted at sites in both the Great Basin and Mojave Deserts. The Great Basin Desert study area consisted of 915 km^2^ of land managed by the United States Department of Defense, United States Army Dugway Proving Ground in west-central Utah. The terrain was typical of Lake Bonneville lakebed characterized by dune systems and alkaline flats that were dominated by black greasewood (*Sarcobatus vermiculatus*). Where wildfires had occurred along the foothills, cheatgrass (*Bromus tectorum*) was common within communities of big sagebrush (*Artemisia tridentata*), rabbitbrush (*Chrysothamnus* spp.), and juniper (*Juniperus osteosperma*) [15]. Elevations across the study area ranged from approximately 1300 to 1800 m. Annual weather consisted of mean air temperatures of 12.69°C (range: −20.02 to 40.58°C) and mean precipitation of 150 mm (MesoWest, Bureau of Land Management & Boise Interagency Fire Center). The US Army Dugway Proving Ground has not been grazed by domestic livestock for the last 60 years [16]. In this study area, we identified 22 permanent water sources consisting of 11 water developments for wildlife, six natural springs, and five man-made ponds. The median distance of 10,000 random points to a natural water source (e.g., springs) and any water source was 7.10 km (range: 0.04 to 19.84 km) and 3.23 km (range: 0.06 to 8.54 km), respectively.

The Mojave Desert study area consisted of 1,064 km^2^ of public land managed by the United States Department of the Interior, Bureau of Land Management. The Mojave study area was located in extreme southwestern Utah, northwestern Arizona, and southeastern Nevada and was approximately 360 km south of Great Basin. This study area was characterized by an alternating landscape of rolling hills/ridges and dry desert washes radiating from the Beaver Dam Mountains and emptying into the Beaver Dam Wash to the southwest near the intersection of the Utah-Nevada-Arizona state borders [21]. In areas that burned within the last decade, red brome (*B. rubens*) was well established among surviving creosote (*Larrea tridentata*), Joshua-tree (*Yucca brevifolia*), and black-brush (*Coleogyne ramosissima*) communities [22]. Along the foothills, the vegetation primarily consisted of sagebrush and juniper, transitioning to pinyon pine (*Pinus edulis*) at higher elevations. Elevations across the Mojave study area ranged from approximately 800 to 2000 m. Annual weather consisted of mean air temperatures of 19.18°C (range: −10.04 to 41.70°C) and mean precipitation of 113 mm (MesoWest, Bureau of Land Management & Boise Interagency Fire Center). The Mojave study area was grazed by livestock from October to May. We identified 66 permanent water sources in this study area consisting of 35 water developments for wildlife, 18 water troughs/tanks for livestock, 11 natural springs, and two man-made ponds. The median distance of 10,000 random points to a natural water source and any water source was 4.75 km (range: 0.03 to 14.01 km) and 2.10 km (range: 0.02 to 8.72 km), respectively.

### Experimental Design and Sampling

To verify if presence of coyotes was greater in areas with free water (hereafter wet) compared to areas without (hereafter dry), we first established wet and dry areas in both study areas. Using ArcGIS (version 10.0, Environmental Systems Research Institute, Redlands, California), we created a uniform pattern of sample points with a distance of 4 km apart for both study areas. Each of these sample points was buffered with a 2.6 km radius based on the square root of a home range for coyotes inhabiting a semi-arid environment similar to our study areas [17]. The square root of the home range is a linear measure used to approximate daily movements of mammals and birds [23], [24]. If free water was located within a buffer zone for a given sample area, we considered it a wet area. We identified water sources using databases with geospatial information for springs and water developments provided by the US Army Dugway Proving Ground and the Utah Division of Wildlife Resources. In addition, we consulted with local ranchers concerning water sources for livestock that were not in our databases. We were confident in our efforts to identify all known water sources in both study areas.

We established 32 scent stations in 2011 and 39 in 2012 and monitored stations for two-week periods during July to August (hottest part of the year; Table 1). Approximately 60% of the scent stations were located in wet areas and 40% in dry areas (Table 1). Scent stations in dry areas were 2.92 km farther from a known water source compared to scent stations in wet areas (Table 1). At each scent station, we placed a scent lure (2011: fatty acid scented disc [Pocatello Supply Depot, Pocatello, Idaho, USA]; 2012: liquid scent [Murray’s Lures, Walker, West Virginia, USA]) on the ground and an infrared-triggered camera (PC 900, Reconyx^©^, Holmen, Wisconsin) approximately two meters from the scent either directly north or south to avoid false camera triggers by the sun.

**Table 1:** Distances from scent stations to nearest known source of free water in wet and dry areas, 2011 to 2012.

Study area	Year	Stations in wet areas		Stations in dry areas	
		Mean distance km (±SE)	*N*	Mean distance km (±SE)	*N*
Great Basin	2011	1.49 (±0.14)	19	4.37 (±0.42)	13
	2012	1.60 (±0.12)	22	4.56 (±0.27)	17
Mojave	2011	1.07 (±0.16)	20	3.82 (±0.33)	12
	2012	0.93 (±0.17)	24	3.74 (±0.29)	15

To evaluate relative rates of visitation to water sources by canids, we monitored all known water sources at the Great Basin and Mojave study areas. From May to October, 2010 to 2012, we used infrared-triggered cameras to photograph canids visiting water sources. We randomly sampled water sources with cameras at both study areas for approximately two-week periods for a total of 78 weeks. To determine which water sources to sample for a given period, we generated random points within each study area using ArcMap. We then identified the nearest water source to a random point and camera-sampled as many water sources as possible (in 2010, we used six cameras in each study area for sampling compared to 15 cameras in 2011–2012). We attached cameras to metal posts and placed them approximately two meters from the edge of water where animals gained access to drink. At water sources with multiple locations of drinking access (e.g., paired tanks of water, ponds), we placed cameras at a minimum of two locations where animals could drink. We considered proximity to trails and recent sign to determine the location of cameras at ponds and large springs [25]. Our estimates of visitation by canids at large water sources were likely conservative due to the inability to monitor all potential locations where canids could access water. We assumed, however, that any potential bias was similar at large water sources from both study areas.

### Statistical Analyses

To test prediction one associated with the indirect effect of water hypothesis, we used *z*-tests for proportions [26] to compare the proportion of scent stations in wet and dry areas visited by canids at both study areas. We tested the spatial segregation component of the second prediction by comparing visits to scent stations by each canid using Kendall’s *Tau-b* correlation analyses. Kendall’s *Tau-b* correlation accounted for ties that occurred because of zero visit data due to only one species of canid primarily visiting a given station [27]. We excluded all stations that were not visited by at least one species of canid from these correlation analyses. To test the temporal segregation component of prediction two, we used time stamps available on photographs of canids at scent stations and compared these times using Mann-Whitney *U*-tests. To account for the transition from 23∶59 to 00∶00 h (midnight) in our analyses and thus avoid the potential difference between photographs taken immediately prior to and after midnight we marked the change between days by setting 21∶00 h (approximately sunset) as 00∶00 h. This adjustment allowed us to compare times of visitation within a continuous scale of time marked by nocturnal hours which are likely more biologically relevant to both coyotes and kit foxes [28], [29].

We used Mann-Whitney *U*-tests to compare the mean daily visitation rates (# of visits/# of operable camera trap days) of canids to water sources between study areas. We defined a visit as all photo captures of a species occurring within 30 min. Thus, photo captures occurring more than 30 min apart were considered independent [30]. We performed all analyses using Program R [31]. We conducted series of statistical analyses to test our hypotheses and we used a Bonferroni correction to avoid type I errors. We set the family-wise level of significance for all statistical tests at α = 0.05 (Bonferroni adjusted α = 0.01).

## Results

**Figure 1:**
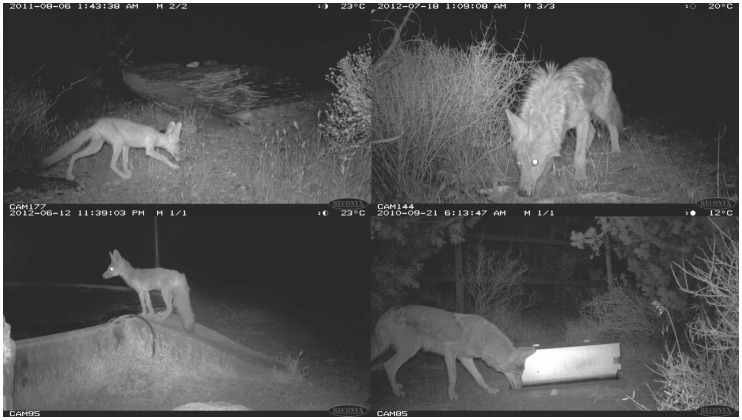
Photo captures of canids from infrared-triggered cameras. Clockwise from top left: kit fox (*Vulpes macrotis*) at a scent station, coyote (*Canis latrans*) at a scent station, coyote at a water development, and kit fox at a water development. Data were collected in the Great Basin and Mojave Deserts, USA, 2011 to 2012.

**Figure 2:**
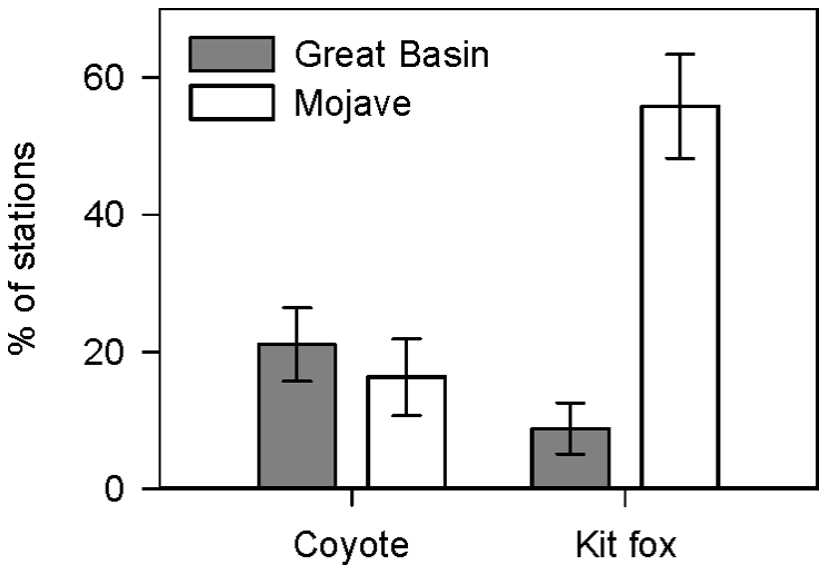
Overall proportions (±SE) of scent stations visited by canids. Data were collected on coyotes (*Canis latrans*) and kit foxes (*Vulpes macrotis*) in the Great Basin and Mojave Deserts, USA, 2011 to 2012.

**Figure 3:**
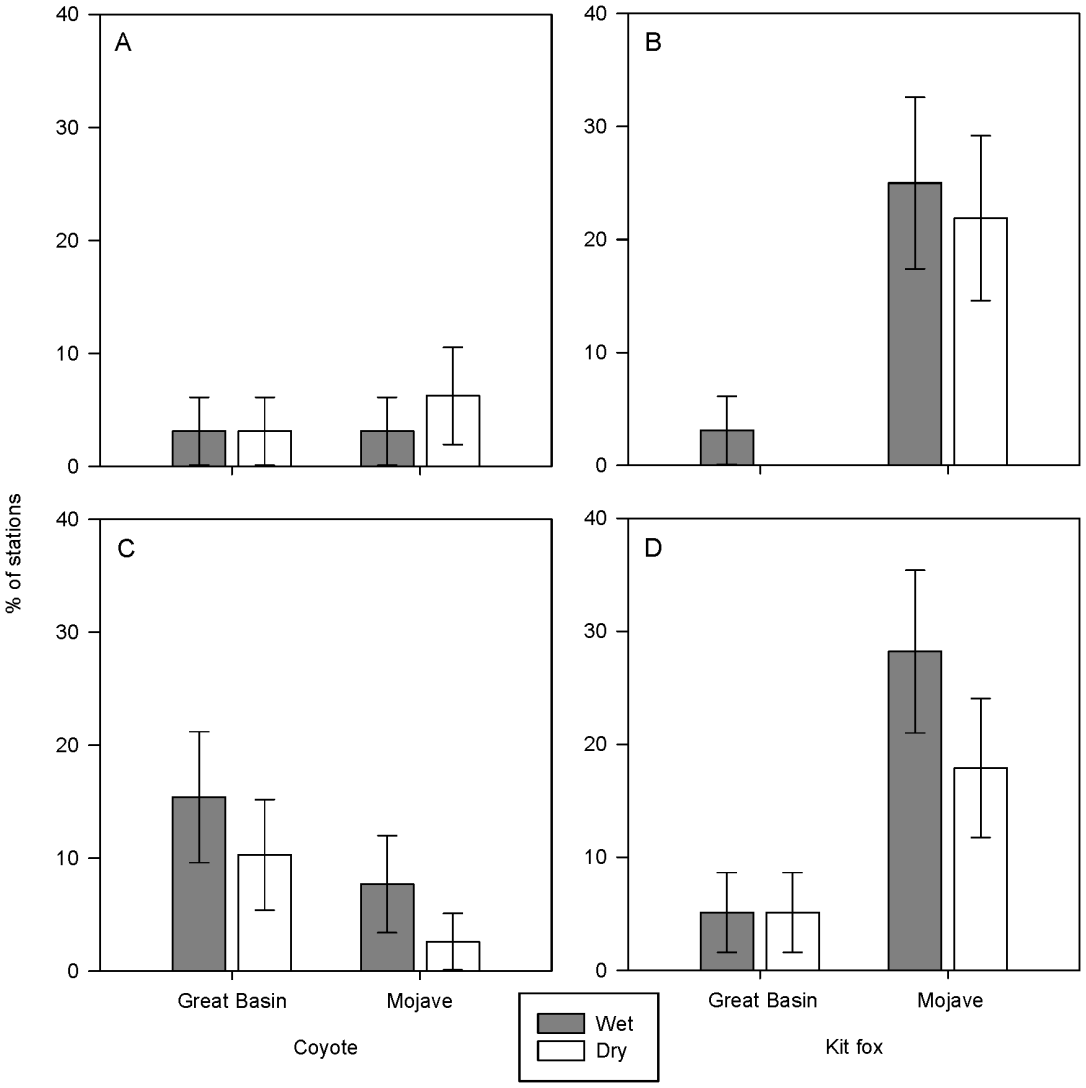
Proportions (±SE) of scent stations visited by canids in wet and dry areas in A) 2011 and B) 2012. Stations in wet areas were ≤2.6 km from free water (mean = 1.25 km) whereas stations in dry areas were ≥2.6 km from free water (mean = 4.17 km). Data were collected on coyotes (*Canis latrans*) and kit foxes (*Vulpes macrotis*) in the Great Basin and Mojave Deserts, USA.

**Figure 4:**
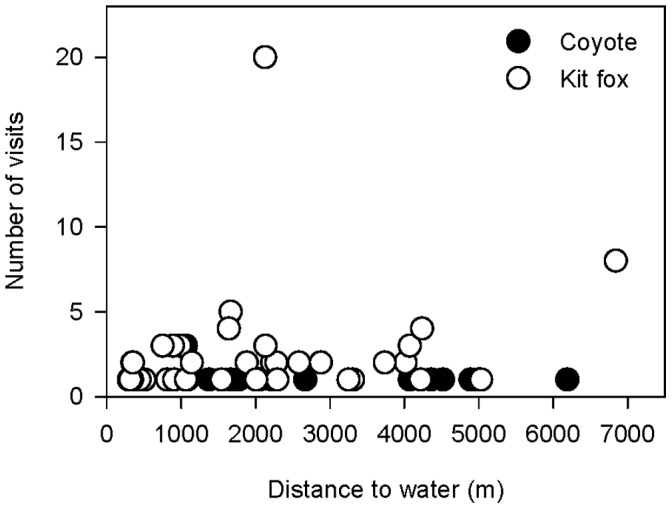
Visitation by canids to scent stations in relation to distance from free water. We defined a visit as all photo captures of a species occurring within 30 min. Data were collected on coyotes (*Canis latrans*) and kit foxes (*Vulpes macrotis*) in the Great Basin and Mojave Deserts, USA, 2011 to 2012.

We detected both coyotes and kit foxes at scent stations in Great Basin and Mojave Deserts (Fig. 1, Dataset S1). Other potential competitors and predators of kit foxes and coyotes that we observed at scent stations included badgers (*Taxidea taxus*), bobcats (*Lynx rufus*), and gray foxes (*Urocyon cinereoargenteus*); however, visits from these intraguild species were too few for meaningful comparison (Table 2). In Great Basin, we detected coyotes at more stations than kit foxes, but in Mojave we found the opposite relationship (Fig. 2). There was no difference between the proportion of stations visited by coyotes in Great Basin or Mojave (2011: *z* = 0.47, *P* = 0.64; 2012: *z* = 1.77, *P* = 0.08; Fig. 2). Conversely, the proportion of stations visited by kit foxes in Mojave was greater than in Great Basin (2011: *z* = 4.04, *P*<0.01; 2012: *z* = 3.52, *P*<0.01; Fig. 2). There was no difference in the proportions of visits to wet and dry stations by coyotes or kit foxes in 2011 (Great Basin coyotes: *z* = 0.28, *P* = 0.78; Great Basin kit foxes: *z* = 0.84, *P* = 0.40; Mojave coyotes: *z* = 1.10, *P* = 0.27; Mojave kit foxes: *z* = 1.01, *P* = 0.31; Fig. 3a) or 2012 (Great Basin coyotes: *z* = 0.27, *P* = 0.79; Great Basin kit foxes: *z* = 0.27, *P* = 0.79; Mojave coyotes: *z* = 0.58, *P* = 0.56; Mojave kit foxes: *z* = 0.05, *P* = 0.96; Fig. 3b). We detected coyotes and kit foxes at scent stations relatively close to and far from free water and did not observe a clear pattern between visitation and distance to free water up to 7 km for either canid (Fig. 4).

**Table 2:** Number of visits to scent stations by species of carnivores in 2011 and 2012.

Species	Great Basin	Mojave	Total
Coyote	13	12	25
Kit fox	11	82	93
Badger	2	1	3
Bobcat	1	1	2
Gray fox	0	2	2

**Figure 5:**
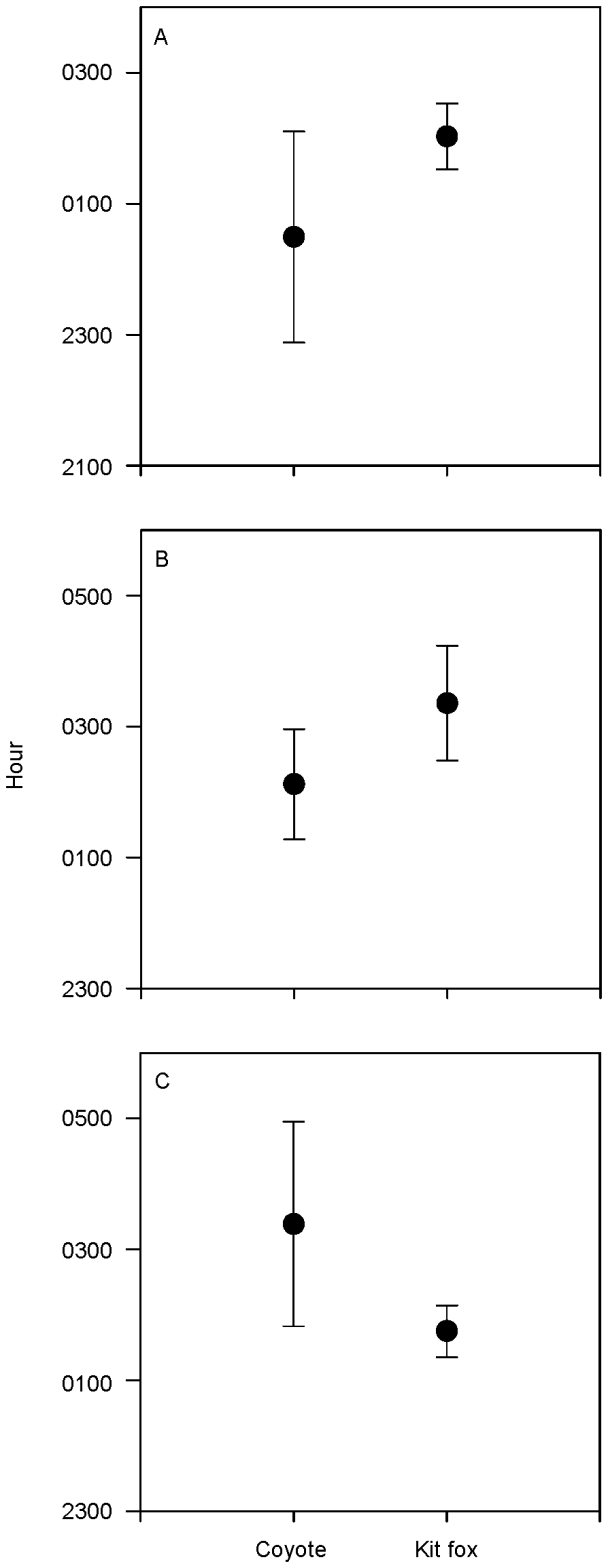
Mean times of day (±SE) when canids visited scent stations. Temporal visitation patterns were observed for coyotes (*Canis latrans*) and kit foxes (*Vulpes macrotis*) in A) Mojave during 2011, B) Great Basin during 2012, and C) Mojave during 2012.

To determine spatial and temporal segregation there were too few visits by canids to scent stations in Great Basin during 2011 for statistical comparison. We did not observe a significant negative correlation between visits of coyotes and kit foxes to scent stations at Great Basin in 2012 (*Tau-b* = −0.68, *P* = 0.02, *df* = 11, *N* = 13). Similarly, in Mojave we did not observe a negative correlation between visits of both canid species during 2011 (*Tau-b* = −0.27, *P* = 0.26, *df* = 15, *N* = 17) or 2012 (*Tau-b* = −0.23, *P* = 0.29, *df* = 17, *N* = 19). There was no difference between the times of day when coyotes and kit foxes visited scent stations in 2011 at Mojave (Mann-Whitney *U* = 63.00, *P* = 0.42; Fig. 5a). In 2012, we did not detect a difference in times when canids visited scent stations in Great Basin (Mann-Whitney *U* = 29.00, *P* = 0.12; Fig. 5b) or Mojave (Mann-Whitney *U* = 118.00, *P* = 0.25; Fig. 5c).

**Figure 6:**
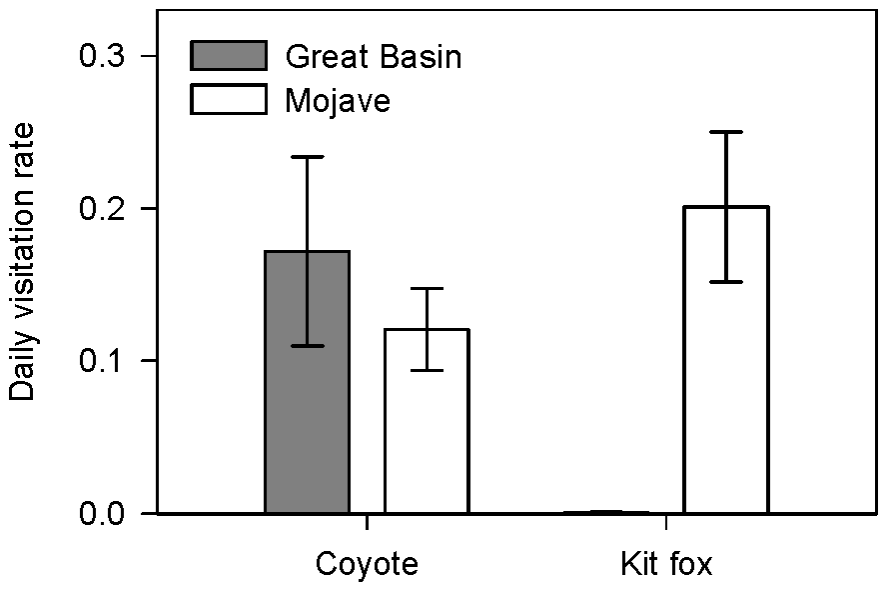
Mean daily visitation rates (±SE) of canids to sources of free water. We defined daily visitation rate as the number of species visits/the number of operable camera trap days per water source. Data were collected on coyotes (*Canis latrans*) and kit foxes (*Vulpes macrotis*) in the Great Basin and Mojave Deserts, USA, 2010 to 2012.

We observed coyotes and kit foxes at water sources in both deserts (Fig. 1, Dataset S2). In 6,476 camera trap days at water sources in Great Basin, we observed 924 coyote visits and four kit fox visits. In 4,803 camera trap days at water sources in Mojave, we observed 353 coyote visits and 1,530 kit fox visits. In Great Basin, coyotes visited 19 of the 22 available water sources whereas kit foxes only visited two. In Mojave, coyotes visited 38 of the 66 available water sources and kit foxes visited 25. There was no difference between mean daily visitation rates for coyotes across study areas (Mann-Whitney *U* = 490.00, *P* = 0.07; Fig. 6). Alternatively, mean daily visitation rate was higher for kit foxes in Mojave than kit foxes in Great Basin (Mann-Whitney *U* = 471.50, *P*<0.01; Fig. 6).

## Discussion

Our study was the first to evaluate the potential indirect effect that anthropogenic water developments may have on canid communities in two deserts. We tested the two predictions of the indirect effect of water hypothesis in the Great Basin and Mojave Deserts. We did not find any support for the first prediction as we detected coyotes equally in wet and dry areas in both deserts (and likewise for kit foxes). Also, we did not find support for the second prediction due to lack of spatial and temporal segregation between coyotes and kit foxes in both study areas. Our data indicated that factor(s) other than the presence or distribution of free water were associated with occurrence of coyotes.

Our spatial scale of inference was restricted to 7 km (the farthest distance between a water source and a scent station) and coyotes can travel farther than 7 km [32]. However, frequent travel beyond the distance of daily movements to visit a water source is energetically costly. If water sources served as a spatial “anchor” and coyotes were liberal in their daily movements, we would have expected to detect more coyotes in areas closer to water relative to areas farther from water. Furthermore, if coyotes were dependent on free water, we would have expected the number of visits by coyotes to scent stations to decrease as the distance of scent stations to the nearest water source increased. Moreover, given the availability of naturally occurring water sources in each area (median distance to water for 10,000 random points was 7.10 and 4.75 km for Great Basin and Mojave, respectively) and the change in availability due to human influence (median distances of 3.23 and 2.10 km for Great Basin and Mojave, respectively) this scale was relevant to both landscapes. Our inference, however, is limited to these scales and we cannot speak to distances beyond 7 km.

Although kit foxes have been observed to spatially and temporally avoid coyotes, results from our scent station experiment did not reveal avoidance [17], [33], [34]. Similar to our observations, other studies have documented kit foxes coexisting with coyotes without avoidance [35], [36]. Observations with other carnivore communities have demonstrated that subordinate competitors can coexist with larger, dominant competitors. For example, coyotes (subordinate) did not spatially adjust their use of habitat to avoid wolves (*C. lupus*), rather coyotes altered behaviors near wolf-killed carcasses [37]. For kit foxes, the availability of resources and refuges (i.e., burrows) likely plays a role in how they partition space and time with coyotes [36], [38].

Our data suggested that kit foxes were less abundant in Great Basin than Mojave, supporting previous reports of reduced populations in Great Basin [15]. However, abundance of coyotes appeared to be similar in both deserts, based on visits to scent stations and water sources. We suggest that coyotes may not solely regulate populations of kit foxes, though they can account for high rates of mortality [39], [40]. Previous work has demonstrated that removal of coyotes did not influence survival of kit foxes, indicating that coyote-induced mortality may be compensatory and that other factors affect population dynamics of kit foxes, such as prey availability [41]–[43].

Historical variation in availability and distribution of free water in western North America may provide, in part, explanation for the lack of support for the indirect effect of water hypothesis in the Great Basin and Mojave Deserts. Western North America has experienced dramatic fluctuations in climate (and associated availability of water) over the last several thousand years. For example, 12,000 y BP much of Great Basin and Mojave was a wetland environment with large lakes [44], [45]. Since that time, this region has alternated between levels of extreme drought and wet conditions [46]. The relatively recent addition of free water (i.e., water developments) in western North America, therefore, may not be novel to species inhabiting this region as both coyotes and kit foxes have experienced these conditions in their evolutionary histories. This natural variation in availability of free water over time has rarely been considered in controversies surrounding anthropogenic modification of water availability [47].

Kit foxes have been perceived to be independent of free water based on physiological and behavioral adaptations [18], [19]. Moreover, historical distributions of kit foxes typically include areas located far from known sources of water, further supporting the notion that this species of canid can exist without free water [48]. Nonetheless, published accounts have reported sporadic use of free water by kit foxes [20], [49]. Our study revealed an extreme rate of visitation to water developments by kit foxes in Mojave not previously reported in other areas of western North America. In Mojave, kit foxes were the most photographed carnivore at water developments and one of the most commonly photographed mammals [21]. The intensity of visitation to water developments by kit foxes in Mojave indicates that arid-adapted species may use water developments more frequently than previously believed [50].

Drinking free water may alleviate physiological stresses and improve survival even for species that are adapted to arid climates [51]. For kit foxes to persist without free water they need to consume nearly twice as much prey per day than what is solely required for energetic demands [18]. By drinking free water, kit foxes may reduce energy and time associated with securing additional prey items to satisfy water demand. Less time spent foraging and less distance traveled in search of prey also reduces the likelihood of encounters with other competitors and potential predators [38]. Furthermore, drinking free water may benefit female kit foxes during lactation due to additional loss of water via production of milk [7]. The frequent visitation to free water by kit foxes in Mojave suggests that water developments may be more beneficial to this species than what has been previously understood [52].

The difference between visitation rates of kit foxes to free water between deserts may be due to at least two factors. First, according to our scent station data, there are likely more kit foxes in Mojave than Great Basin, resulting in an increased probability of detection at free water. Second, nighttime temperatures at Great Basin (mean = 14.42°C, SE = 0.09) were on average 8.70°C cooler than Mojave (mean = 23.12°C, SE = 0.08) although maximum daytime temperatures at both study areas were similar (∼41°C). Relatively warmer nighttime temperatures in Mojave likely resulted in a reduced thermal gradient for kit foxes to dissipate heat from nocturnal activities which may have resulted in higher rates of water loss [19] compared to kit foxes in Great Basin. Relatively warmer nights and subsequent higher rates of water loss likely created a greater physiological demand for free water by kit foxes in Mojave compared to Great Basin.

Our results did not provide support for the indirect effect of water hypothesis at Great Basin or Mojave Deserts. The apparent high abundance of kit foxes that we observed in Mojave, an arid landscape with many water developments, created a paradox based on the logic that water developments indirectly influence these canids via increased distribution of coyotes [15], [16]. We did not find any support for the assertion that free water played a negative indirect role on kit foxes. Furthermore, our data did not indicate partitioning of activity in space or time. We reject the indirect effect of water hypothesis as operational in our study areas during our study years at the spatial scale evaluated. The ultimate factor(s) that influence the distribution of coyotes and kit foxes in these two deserts are unknown and warrant further study.

## Supporting Information

Dataset S1 **All visits to scent stations recorded by infrared-triggered cameras.** We defined a visit as all photo captures of a species occurring within 30 min. Data were collected in the Great Basin and Mojave Deserts, USA, 2011 to 2012.
(XLSX)

Dataset S2 **All visits to water sources recorded by infrared-triggered cameras.** We defined a visit as all photo captures of a species occurring within 30 min. Data were collected on coyotes (*Canis latrans*) and kit foxes (*Vulpes macrotis*) in the Great Basin and Mojave Deserts, USA, 2011 to 2012.
(XLSX)
